# Supplementary material for: The methylation status of the embryonic limb skeletal progenitors determines their cell fate in chicken
Source: Commun Biol. 2020 Jun 5;3:283. doi: 10.1038/s42003-020-1012-3 (PMC7275052; doi:10.1038/s42003-020-1012-3)
Supplement: Supplementary file 2 — Reporting Summary [file 42003_2020_1012_MOESM2_ESM.pdf]

nature research

Corresponding author(s): Juan M. Hurlé and Juan A. Montero.

Last updated by author(s): May 11, 2020

Reporting Summary

Nature Research wishes to improve the reproducibility of the work that we publish. This form provides structure for consistency and transparency in reporting. For further information on Nature Research policies, seeAuthors & Referees and theEditorial Policy Checklist .

Statistics

For all statistical analyses, confirm that the following items are present in the figure legend, table legend, main text, or Methods section.

n/a	Confirmed	
☐	☒	The exact sample size (*n*) for each experimental group/condition, given as a discrete number and unit of measurement
☐	☒	A statement on whether measurements were taken from distinct samples or whether the same sample was measured repeatedly
☐	☒	The statistical test(s) used AND whether they are one- or two-sided<br>*Only common tests should be described solely by name; describe more complex techniques in the Methods section.*
☒	☐	A description of all covariates tested
☒	☐	A description of any assumptions or corrections, such as tests of normality and adjustment for multiple comparisons
☒	☐	A full description of the statistical parameters including central tendency (e.g. means) or other basic estimates (e.g. regression coefficient) AND variation (e.g. standard deviation) or associated estimates of uncertainty (e.g. confidence intervals)
☐	☒	For null hypothesis testing, the test statistic (e.g. *F*, *t*, *r*) with confidence intervals, effect sizes, degrees of freedom and *P* value noted<br>*Give P values as exact values whenever suitable.*
☒	☐	For Bayesian analysis, information on the choice of priors and Markov chain Monte Carlo settings
☒	☐	For hierarchical and complex designs, identification of the appropriate level for tests and full reporting of outcomes
☒	☐	Estimates of effect sizes (e.g. Cohen's *d*, Pearson's *r*), indicating how they were calculated

Our web collection on statistics for biologists contains articles on many of the points above.

Software and code

Policy information about availability of computer code

Data collection	Stereomicroscope Nikon SMZ1500 with Digital Camera DXM1200C and software ACT-1C; Laser Confocal Microscope Zeiss LSM510 with LSM510 software; Cytoflex (Beckman Coulter) with Cytexpert Software; Odyssey Infrared-imaging system Li-Cor Biosciences; ELISA Methylation Multiskan Ex Thermo with ASCENT software v2.6 ; MSRE-QPCR StepOne RT System with StepOne software v2.3; QPCR Stratagene Mx3005P system with MxPro software v4.10.
Data analysis	GraphPad Prism 5.

For manuscripts utilizing custom algorithms or software that are central to the research but not yet described in published literature, software must be made available to editors/reviewers. We strongly encourage code deposition in a community repository (e.g. GitHub). See the Nature Research guidelines for submitting code & software for further information.

Data

Policy information about availability of data

All manuscripts must include a data availability statement. This statement should provide the following information, where applicable:
- Accession codes, unique identifiers, or web links for publicly available datasets
- A list of figures that have associated raw data
- A description of any restrictions on data availability

Data are available in the manuscript and on request from the authors.

Field-specific reporting

Please select the one below that is the best fit for your research. If you are not sure, read the appropriate sections before making your selection.

Life sciences study design

All studies must disclose on these points even when the disclosure is negative.

Sample size	Sample sizes were determined on the basis of the variability of the different performed measures:<br>QPCR n>5; Western Blot n=4; Flow Cytometry n>6; Methylation ELISA n>3; MSRE-QPCR n>3; IHS & Histological experiments n>6.
Data exclusions	No data were excluded.
Replication	All the measurements included at least three independent experiments performed under the same conditions. These replication attempts were successful.
Randomization	n/a
Blinding	n/a

Reporting for specific materials, systems and methods

We require information from authors about some types of materials, experimental systems and methods used in many studies. Here, indicate whether each material, system or method listed is relevant to your study. If you are not sure if a list item applies to your research, read the appropriate section before selecting a response.

Materials & experimental systems

n/a	Involved in the study	
☐	☒	Antibodies
☒	☐	Eukaryotic cell lines
☒	☐	Palaeontology
☐	☒	Animals and other organisms
☒	☐	Human research participants
☒	☐	Clinical data

Methods

n/a	Involved in the study	
☒	☐	ChIP-seq
☐	☒	Flow cytometry
☒	☐	MRI-based neuroimaging

Antibodies

Antibodies used	anti-DNMT1 (NB100-264; NovusBio.Co. USA); anti-DNMT3a (ab2850 and ab188479; Abcam); anti-DNMT3b (ab2851; Abcam); OptimAb anti-5-methylcytosine (33D3; BI-MECY-0500 Eurogentec); anti-MDC1 (Ab41951; Abcam); anti-phospho-histone H2A.X (Ser139) (JBW301; 05-636; Sigma-Aldrich, Millipore-Upstate) and anti-Sox9 (AB5535; Sigma-Aldrich; Merck-Millipore).
Validation	All antibodies are commercially available and have been previously tested in our research model. Routine controls were performed by suppressing the primary antibody.

Animals and other organisms

Policy information about studies involving animals; ARRIVE guidelines recommended for reporting animal research

Laboratory animals	Rhode Island chicken embryos from day 4 to 8.5 of incubation (id) equivalent to stages 23 to 34HH.
Wild animals	The study did not involve wild animals.
Field-collected samples	The study did not involve samples collected from the field.
Ethics oversight	All experiments were carried out under the standards of experimentation dictated by the European Community (2010/63 / EU) in accordance with Spanish legislation (RD53 / 2013) and approved by the ethics committee of the local government through the authorization corresponding to our research project (PI-03-18).

Note that full information on the approval of the study protocol must also be provided in the manuscript.

Flow Cytometry

Plots

Confirm that:

- [ ] The axis labels state the marker and fluorochrome used (e.g. CD4-FITC).
- [ ] The axis scales are clearly visible. Include numbers along axes only for bottom left plot of group (a 'group' is an analysis of identical markers).
- [ ] All plots are contour plots with outliers or pseudocolor plots.
- [x] A numerical value for number of cells or percentage (with statistics) is provided.

Methodology

Sample preparation	As indicated in the manuscript, cell death was evaluated by flow cytometry in dissociated cultured cells after the functional experiments. One million cells were used in each test. For propidium iodide (PI) staining, the cells were washed with PBS and fixed in 90% ethanol. The samples were incubated overnight at 4oC with 0.1% sodium citrate, 0.01% Triton X-100 and 0.1 mg/ml PI.
Instrument	Cytoflex (Beckman Coulter)
Software	Cytexpert Software
Cell population abundance	The abundance of living and apoptotic cells is one of the objectives of the experiment and its results are included in the manuscript
Gating strategy	The gating strategy has been performed by deselecting impurities (debris) employing an SSC-A / IP-PE dot plot and then selecting the apoptotic and living cells in a logarithmic scale IP-PE histogram. Finally, we quantify the cell cycle phases of living cells in a linear scale IP-PE histogram.

- [x] Tick this box to confirm that a figure exemplifying the gating strategy is provided in the Supplementary Information.
