# Supplementary material for: The methylation status of the embryonic limb skeletal progenitors determines their cell fate in chicken
Source: Commun Biol. 2020 Jun 5;3:283. doi: 10.1038/s42003-020-1012-3 (PMC7275052; doi:10.1038/s42003-020-1012-3)
Supplement: Supplementary file 3 — Supplementary Information [file 42003_2020_1012_MOESM3_ESM.pdf]

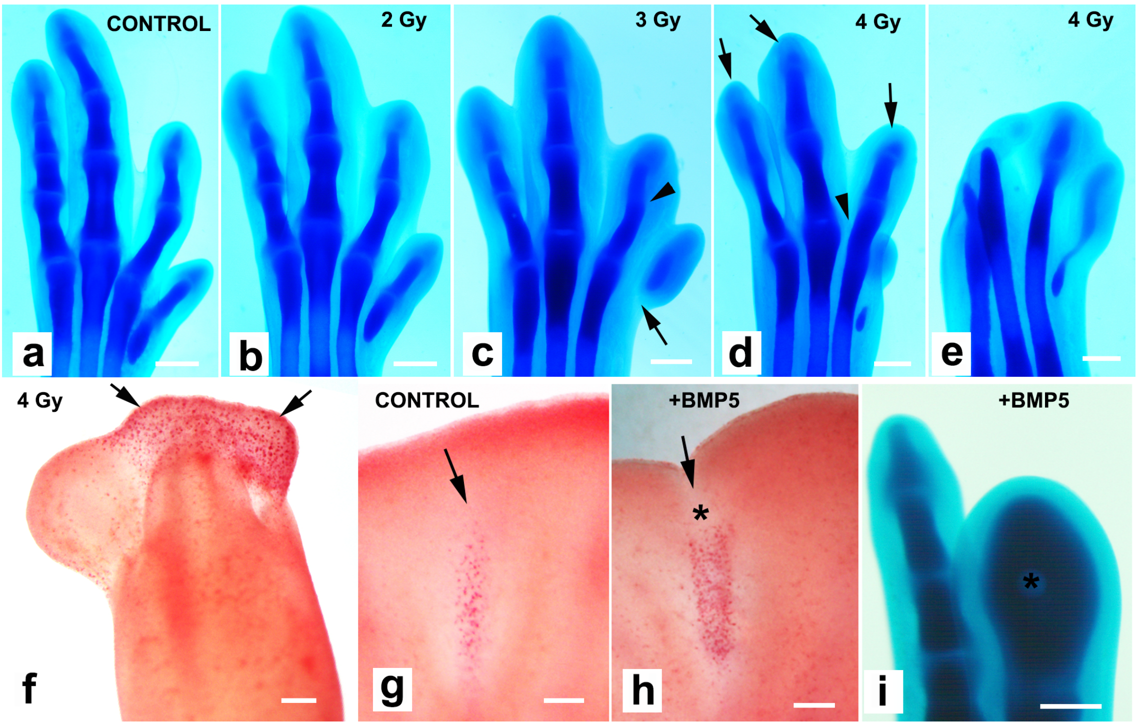

Supplementary figure 1

a-e, morphology of the autopod skeleton stained with alcian blue in control **(a)** and 3 days after embryo irradiation at doses of 2Gy **(b),** 3Gy **(c),** and 4Gy **(d-e).** Note the absence of alterations in **b**, contrasting with the presence of joint inhibition (arrow head) and digit truncation (arrow) after irradiation at 3Gy **(c). d** and **e**, are mild **(d)** and severe **(e)** skeletal phenotypes of embryos irradiated at a dose of 4 Gy. Arrows show digit truncations and arrow heads indicate missing joints. **f**, shows a severely malformed autopod, 3 days after irradiation with 4Gy, vital stained with neutral red, showing the massive cell death process (arrows) that remove undifferentiated autopodial progenitors. **g-h**, control **(g)** and BMP-treated **(h)** interdigits 30hr after the implantation of a bead (*) soaked in 5µg/ml BMP5 showing the pattern of cell death (arrows) by neutral red vital staining. **i**, enlargement of the tip of digit 3, three days after implantation of a BMP5-bead (*). Bars=200µm

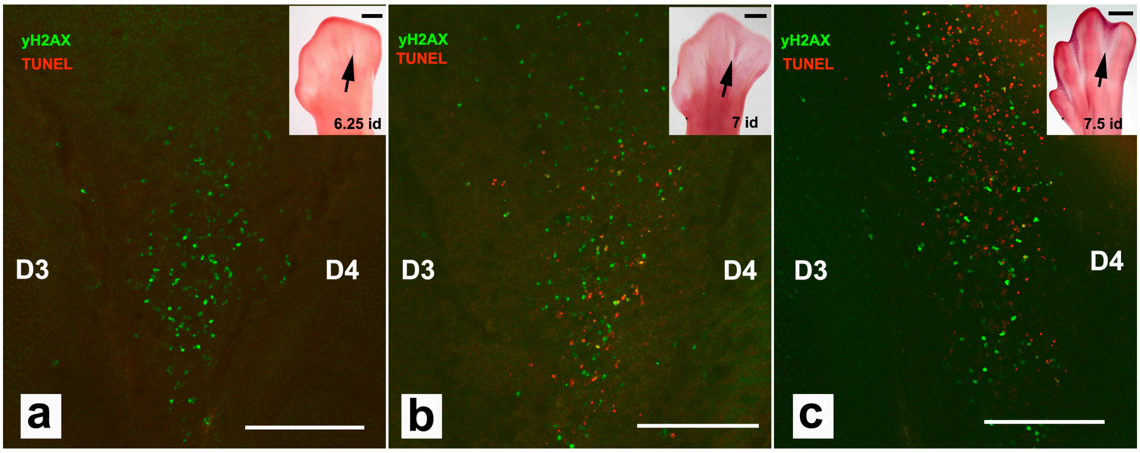

Supplementary figure 2

a-c.- Double labeling with γH2AX and TUNEL of interdigit tissue sections of autopods at id 6.25 (**a,** HH stage 29-30), 7 (**b;** HH stage 31) and 7.5 (**c;** HH stage 32) to illustrate the normal course of degeneration during tissue remodeling. The insets illustrate the gross morphology of the autopods at the same stages vital-stained with neutral red. Arrows indicate the interdigit region analyzed in the confocal images. Note that γH2AX labeling (green) is the predominant degenerative feature at the beginning of tissue remodeling while TUNEL labeling (red) predominates at advanced stages of tissue remodeling. Bars=250µm

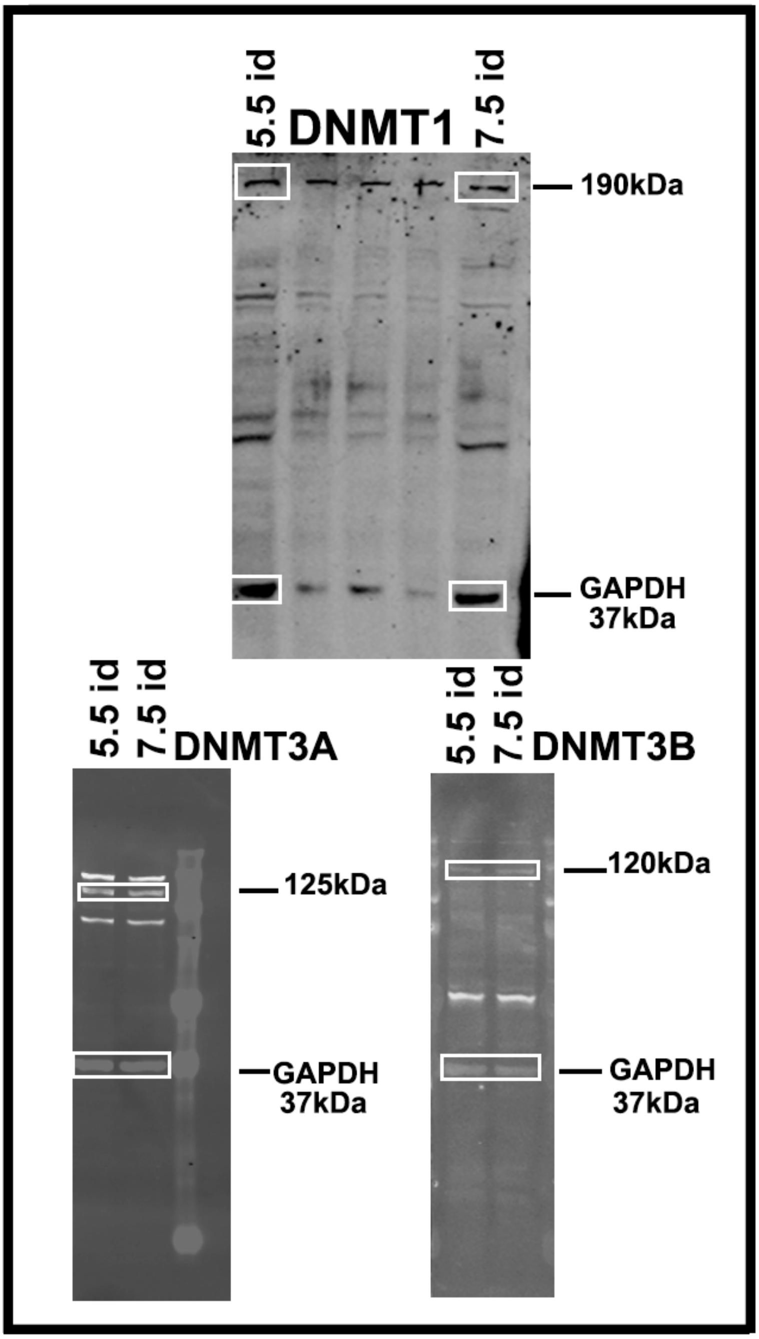

Supplementary figure 3

Source data for Fig.6 a-c in the manuscript.

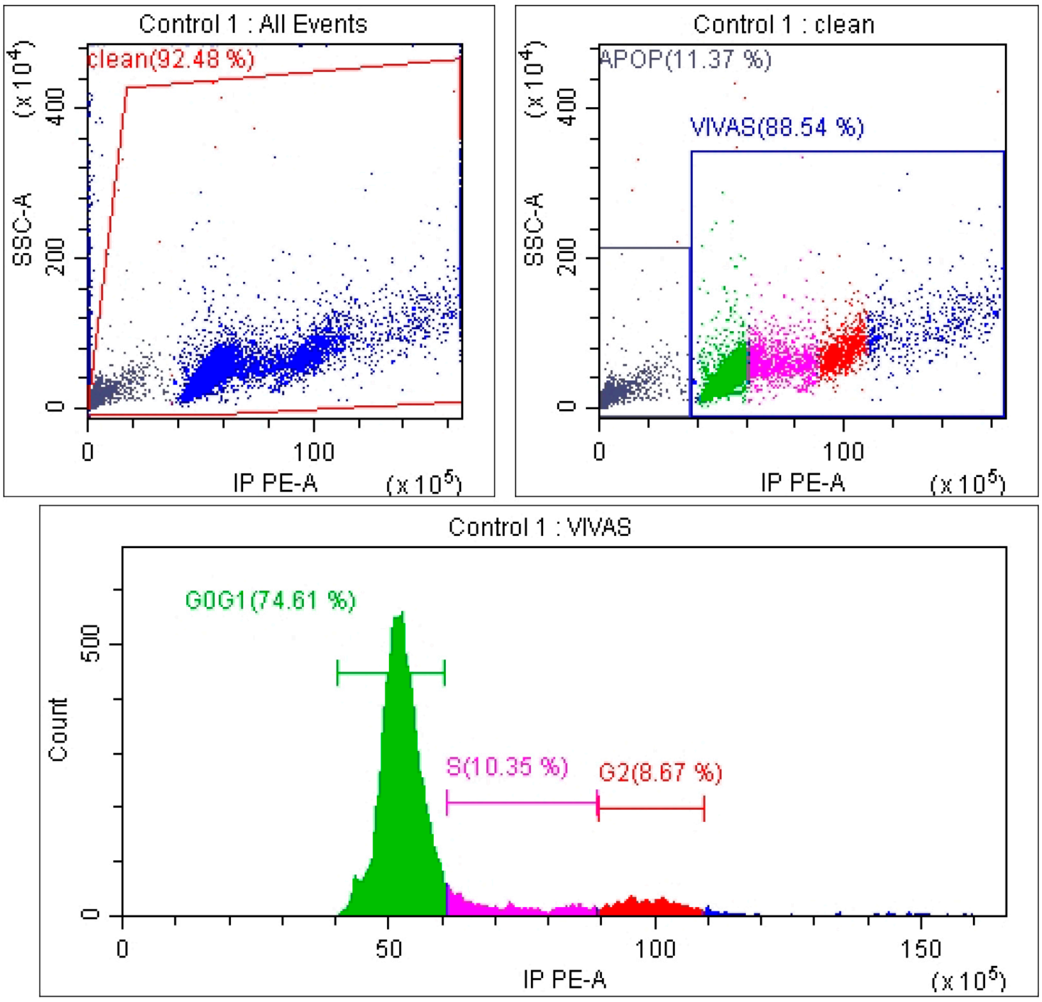

Tube Name: Control 1

Sample ID:

Population	Events	% Total	% Parent
All Events	12212	100.00 %	100.00 %
clean	11294	92.48 %	92.48 %
APOP	1284	10.51 %	11.37 %
VIVAS	10000	81.89 %	88.54 %
G0G1	7461	61.10 %	74.61 %
S	1035	8.48 %	10.35 %
G2	867	7.10 %	8.67 %

Supplementary figure 4

Figure exemplifying the gating strategy reported in the manuscript in flow cytometry experiments. The figure illustrates the analysis of a control population of cells in our experiments.
